# Cognitive Restructuring and Graded Behavioural Exposure for Delusional Appraisals of Auditory Hallucinations and Comorbid Anxiety in Paranoid Schizophrenia

**DOI:** 10.1155/2014/124564

**Published:** 2014-09-11

**Authors:** Pawel D. Mankiewicz, Colin Turner

**Affiliations:** ^1^National Health Service, South Essex Partnership University NHS Foundation Trust, Psychosis and Complex Mental Health Specialist Interest Group, Disability Resource Centre, Poynters House, Poynters Road, Dunstable LU54TP, UK; ^2^National Health Service, Lincolnshire Partnership NHS Foundation Trust, Adult Mental Health Rehabilitation Specialty, Discovery House, Long Leys Road, Lincoln LN11FS, UK

## Abstract

The prevalence of diagnostic comorbidity between psychosis and anxiety disorders has been found to be considerable. Cognitive models of psychosis suggest that anxiety does not arise directly from positive symptoms of schizophrenia but rather from an individual interpretation of such experiences. In the United Kingdom, cognitive-behavioural therapy for psychosis (CBTp) has been recommended within clinical guidelines as a psychological treatment of choice for those diagnosed with schizophrenia. However, despite empirical evidence supporting CBTp, the treatment provision remains infrequent and not routinely available. This case describes a successful implementation of CBTp. Sixteen sessions were delivered to a 40-year-old male with diagnoses of paranoid schizophrenia and comorbid anxiety, focusing primarily on cognitive restructuring of paranoid appraisals of auditory hallucinations and behavioural experiments employed progressively via graded exposure to anxiety-inducing stimuli. Standardised measurements, behavioural frequency sampling, and subjective data indicated a considerable reduction in both paranoia and anxiety. Also, the client's psychosocial functioning improved substantially. This report indicates that the treatment may help those with experiences of psychosis and comorbid anxiety reach a significant improvement in their quality of life and offers an encouraging and innovative perspective on direct engagement with the content of paranoia and voices at the onset of therapy.

## 1. Introduction

The specific causes of psychosis-type experiences remain unclear [1]. Although, since the inception of the term of schizophrenia, numerous hypotheses were proposed, few of them still uphold some scientific validity. Generally, the outcomes of the nature-nurture debate in professional literature might be concluded with reflection that a range of factors relating to the areas of psychological makeup of the individual, the person's environment, and biological background interact with each other and may all contribute to the development of such experiences [2].

For instance, the gene-stress interaction hypothesis suggests that prolonged exposure to psychosocial distress (e.g., childhood traumas, life events, and discrimination) may with time contribute to sustained dysregulation of the hypothalamic-pituitary-adrenal axis leading to dopamine sensitization in mesolimbic areas and increased stress-induced striatal dopamine release; individual vulnerability to such neurochemical change is proposed to be genetically influenced [3]. Alternatively, a neurocognitive hypothesis of inner speech proposes that some individuals, due to neurochemical deficits in self-monitoring, may eventually experience incidents of inability to recognise inner speech as self-produced and, instead, appraise such speech as autonomous, outer voice [1]. An impaired self-monitoring has also been found in behavioural and neuroimaging studies among individuals with delusions [4].

Furthermore, it has been widely acknowledged that persons with psychosis present with much higher rates of alcohol and illicit drug misuse than the general population, and such misuse may inevitably contribute to increased severity of symptoms [5]. For example, cannabis has been recognised as a psychoactive substance that not only may induce psychosis and anxiety [6], but also may exacerbate the symptoms and increase anxious responses to such symptoms [7] through the agonistic effects on cannabinoid receptors among patients already diagnosed with schizophrenia. Likewise, alcohol use disorder has been hypothesised to effect symptoms of psychosis and comorbid dysphoria through its transient effects on multiple neurotransmitter systems and is characterised with the consequent gradual decline in the general psychosocial functioning, poor adjustment, and bad treatment outcomes among individuals with diagnoses of schizophrenia [8].

The cognitive-behavioural approach to psychosis describes psychotic phenomena through the underlying cognitive, emotional, and behavioural processes, which are hypothesised to constitute a psychological aftermath of distressing, often overwhelming, depriving, and traumatic experiences [9]. Given that similar psychosocial aetiological factors have been recognised in depression and anxiety, it does not seem surprising that epidemiological studies demonstrated a considerable incidence of mood disorders among individuals with diagnoses of paranoid schizophrenia [10]. The prevalence of diagnostic comorbidity was found to be as high as 57.3%, of which approximately 62% of people were found to have some form of anxiety disorder [11].

Evidence suggests that experiencing positive symptoms of psychosis, particularly auditory hallucinations, as dominating, insulting, and commanding correlates with higher levels of psychological distress [12]. Norman and Malla [13] reported that high levels of anxiety were associated with hallucinations and delusions, but not with negative symptoms. Also, studies reviewed by Bentall [14] suggested that positive symptoms of psychosis, such as paranoid ideations, were accompanied by emotional distress, including anxiety. Cognitive-behavioural treatment models of psychosis propose that anxiety does not arise directly from positive symptoms of psychosis but rather from an individual interpretation of those symptoms and personal meanings attached to such experiences [15]. Thus, the appraisal of unusual phenomena appears to play the critical role in determining whether or not an individual arrives at a paranoid interpretation of hallucinatory experiences. Simultaneously, the cognitive content of distress is hypothesised to express itself in a symptom of psychosis, such as a persecutory delusion, which in turn directly contributes to the exacerbation of emotional distress; for instance, anxiety will arise from the belief of threat [16]. Furthermore, current evidence suggests that paranoid ideation and psychoticism, if accompanied by high levels of psychological distress, may considerably decrease one's subjective wellbeing and life satisfaction [17]. Since emotional distress has been shown not only to precipitate and follow the positive symptoms of psychosis but also to contribute to the immobilizing experience of such symptoms, it has been argued that the primary target of cognitive-behavioural therapy for psychosis (CBTp) should not be the presence of hallucinations or delusions, but rather the emotional distress they cause to an individual and perceived inability to cope with such experiences [18].

In the past decade empirical investigations of CBTp have flourished and the results of studies examining the effectiveness of CBTp appear encouraging. In their meta-analytical evaluation of controlled research and qualitative reviews, Roth and Fonagy [19] discussed numerous studies with favourable outcomes. CBTp has also been supported with favourable results of case series [20] and randomised control trials [21]. An RCT conducted by Tarrier et al. [22] demonstrated that both positive symptoms of psychosis and accompanying emotional distress reduced considerably over the course of CBTp. Furthermore, specific assumptions of cognitive-behavioural models of psychosis have been subjected to empirical enquiries. For instance, as shown by Luzón et al. [23], individuals with active psychosis reporting catastrophic interpretations of their experiences exhibited elevated anxiety levels.

In the United Kingdom, the growing evidence supporting CBTp affected the recommendations made by the National Institute for Health and Care Excellence in their clinical guideline for schizophrenia. In 2009, the guideline recommended CBTp as one of the core interventions for adults with psychosis [24]. Such recommendation was upheld in the most recent revision of the guideline [25]. CBTp should now be offered to all individuals with diagnoses of schizophrenia and delivered over a minimum of 16 individual sessions. However, despite national policies, numerous studies revealed that CBTp was still not routinely available to patients and its delivery appeared sporadic and unsatisfactory [26]. Those with acute episodes of psychosis on inpatient mental health wards seemed particularly disadvantaged in terms of CBTp provision and only as few as 3.9% of eligible inpatients participated in the recommended treatment [27].

In order to put the above into the context of clinical practice, we will consider the case of “Raymond,” who received a full course of CBTp in line with the clinical guidelines in the UK. This case provides the opportunity for reflection on the effectiveness of cognitive-behavioural treatment in schizophrenia, with particular focus on the interventions of cognitive restructuring and graded behavioural exposure, delivered to an individual with a longstanding history of auditory hallucinations, paranoia, and comorbid anxiety. Raymond has consented for the case study to be written and used for educational and publishing purposes. A pseudonym has been used to protect the client's identity.

## 2. Case Presentation

### 2.1. Case History and Presenting Problems

Raymond was a 40-year-old male referred to a specialist service for adults with psychosis and complex mental health needs in one of the National Health Service Trusts in East Midlands, UK, for an individual psychotherapeutic input. Raymond was diagnosed with paranoid schizophrenia and a comorbid anxiety disorder (not otherwise specified). A number of ongoing symptoms were reported, including derogatory and threatening auditory hallucinations, paranoid delusions, high anxiety levels, and social avoidance and withdrawal. Past and ongoing interventions consisted of pharmacotherapy with a maintenance dose of antipsychotics and tranquilisers, social inclusion activities facilitated by a community based team, and recurrent crisis oriented admissions to acute mental health wards.

Since early adolescence, Raymond regularly used excessive amounts of alcohol and cannabinoids, which initially seemed to be his way of conforming to peer pressure in the deprived area where he lived. He received strict upbringing from his father; thus spending hours in pubs appeared to function as an avoidance of exposure to distressing stimuli at home. With time, Raymond became dependent on the use of illicit substances. He was trained as a builder and enjoyed his work. Yet, after the onset of psychosis Raymond gave up his trade. His first episode of hearing voices occurred at the age of 30 and involved his first admission to an acute mental health ward, where he underwent an alcohol detoxification. Raymond has managed to remain abstinent from alcohol since and yet continued to use cannabinoids on a regular basis. After a few years of remission, the second episode of psychosis occurred and was followed by another inpatient admission. Subsequently, Raymond remained abstinent from cannabis as well. However, auditory hallucinations persisted on a daily basis. Additionally, Raymond developed a range of paranoid appraisals of voices and delusional beliefs about other people's vicious intentions towards him, which precipitated social withdrawal and triggered high anxiety levels. At the time of referral, Raymond lived isolated on his own in a house, where he had installed surveillance cameras and barricaded his bedroom at nights. He was unemployed and in receipt of social benefits.

### 2.2. Case Assessment and Measures

As in generic cognitive-behavioural models, assessment in CBTp aims to evolve into a case formulation; hence a range of cognitive interview methods were employed. Furthermore, to formally assess the person's symptomatic presentation and evaluate the intervention outcomes, a standardised psychiatric measure, the Brief Symptom Inventory (BSI), was administered with the client. The BSI is a 53-item self-report inventory, which has been designed to reflect the symptom patterns among mental health in- and outpatients. Each BSI item is rated on a five-point scale (0–4) reflecting a person's distress from “not at all” to “extremely.” The BSI is a measure of the current symptom status and is scored on the following subscales: somatisation, obsessive-compulsive, interpersonal sensitivity, depression, anxiety, hostility, phobic anxiety, psychoticism, and paranoid ideation [28]. The BSI was shown to demonstrate sufficient psychometric properties. Normative samples for BSI included 1002 adult psychiatric outpatients, 974 adult nonpatients, 423 adult psychiatric inpatients, and 2408 adolescent nonpatients; internal consistency was established using Cronbach's alpha coefficients for all nine dimensions, which ranged from 0.71 to 0.85, while test-retest reliability coefficients were estimated between 0.68 and 0.91 [29]. Internal structure and construct validity were found to be adequate: orthogonal varimax loadings determined from principal components analysis ranged from 0.35 to 0.71 [29]. Convergent and discriminant validity was examined through comparison with the* Minnesota Multiphasic Personality Inventory*; correlation coefficients scoped from 0.31 to 0.72 [29]. The BSI has also been standardised and normalised on the British population [30, 31].

For the purpose of Raymond's assessment, the BSI subscales of paranoid ideation (PAR) and anxiety (ANX) were administered. Raymond's preintervention PAR score was 2.20 and his ANX score was 2.50. Both scores were elevated by more than one standard deviation above the UK outpatient mean and indicated heightened levels of difficulties in both symptom areas.

### 2.3. Formulation

**Figure 1:**
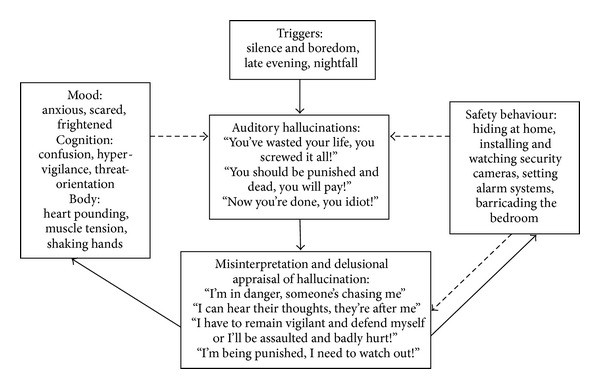
Diagrammatic depiction of case formulation, based on Morrison et al. [9].

As depicted in Figure 1, a cognitive model of psychosis with comorbid emotional distress and safety behaviour developed by Jones [1] was employed to formulate Raymond's symptomatic experiences and to illustrate his difficulties in a diagrammatic form.

During the assessment, Raymond identified a number of triggers. He noticed that he would hear voices while he was bored at home, kept silent, or had nothing to occupy his mind with. Furthermore, the voices would become particularly active in the late evening, upon nightfall. The voices felt like they were coming from inside of his head and were screaming derogatory and threatening comments. Raymond misinterpreted the voices as hearing someone else's thoughts and was becoming increasingly delusional in his beliefs about other people, including his neighbours and random pedestrians. In consequence, he experienced elevated emotional, cognitive, and bodily symptoms of anxiety and employed a range of safety behaviours. These, in turn, contributed to his hypervigilance and preoccupation with the voices and prevented disconfirmation of his paranoid beliefs.

Based on the recommendations made in the clinical guideline for schizophrenia, 16 sessions of individual CBTp were contracted. The following intervention goals were agreed on: enhancement of strategies to cope with voices, paranoid/delusional beliefs and anxiety, and reestablishment of autonomy at nights. The intervention plan was informed by a CBTp manual [32] and incorporated the following treatment modules: psychoeducation, cognitive restructuring of delusional appraisals of voices, behavioural training (graded exposure), cognitive therapy for secondary symptoms (comorbid anxiety), and self-management planning (relapse prevention).

### 2.4. Psychoeducation

Based on the formulation diagram, the cognitive model of psychosis was thoroughly discussed. Raymond was educated about the significance of cognitive mediation in psychosis, where particular appraisals of voices predict individual distress and coping behaviour [33]. In Raymond's case, persecutory beliefs triggered high anxiety levels, avoidance, and escape-type reactions. The crucial role of safety behaviour in maintaining anxiety and preventing the disconfirmation of delusions [34] was also discussed.

Furthermore, the concept of “punishment paranoia” [35] as a cognitive representation of fundamental concerns in one's life was brought to Raymond's attention. Also, relevant outcomes of cognitive neuroscience research in auditory hallucinations were discussed, particularly, the evidence for subvocalisation that accompanies the experience of hearing voices, suggesting that auditory hallucinations might be misattributions of internal mental events [36].

### 2.5. Cognitive Restructuring of Delusional Appraisals of Voices

In order to compassionately challenge and restructure the paranoid appraisals of auditory hallucinations, this module of the treatment began with evidential analysis of the content of delusional beliefs [37]. Guided discovery technique was regularly used with good effects. Raymond found no evidence to support his persecutory beliefs about voices. Contrary to his appraisals, Raymond realised that he had remained safe and had not been attacked or even threatened by anyone for numerous years.

Once the initial doubt in delusional explanations was instigated, cognitive restructuring proceeded to the reattribution of beliefs about voices [9]. In this stage, Raymond recognised the presence of recurrent negative internal dialogues he conducted with himself in his thoughts, which frequently precipitated the experiences of auditory hallucinations. The voices, in turn, seemed to represent his essential worries about his life, for example,* “I've wasted my life.”* Subsequently, Raymond made a pragmatic use of psychoeducational discussions on the subjects of misattribution of internal mental events and punishment paranoia. He developed an understanding of functional associations between voices, persecutory cognitions, and his own concerns and expressed his disappointment with how he had conducted his life since adolescence, hence incorporating the new knowledge he had gained so far in therapy.

Subsequently, Raymond was encouraged to practice identification of internal dialogues on a daily basis. Such dialogues occurred mainly in the moments of boredom. To restructure such unhelpful and dysfunctional cognitive experiences, Raymond begun implementing a range of modified self-statements [34], which were initially agreed on with the therapist, for example,* “I do not need to be punished for anything, as I have never hurt anyone. I have already improved my life, quit drinking and drugs, and deserve to be happier.”* From this point in therapy, Raymond regularly practiced reframing the persecutory appraisals of his experiences, identifying anxiety-inducing cognitions and replacing them with evidence and modified self-statements.

### 2.6. Behavioural Training (Graded Exposure)

Following completion of the cognitive restructuring module, Raymond reported a noticeable reduction in his experiences of anxiety. Subsequently, he voiced a growing readiness to relax a range of his safety behaviours he employed predominantly in the night time. Hence, behavioural experiments were used to address the unhelpful behaviour maintaining the cycle of paranoid appraisals of voices and comorbid emotional distress [38]. The overarching assumption tested was “*If I do not remain isolated and vigilant, activate the security system and barricade the bedroom, then I'll be assaulted.”* Initially, testing such assumption appeared too challenging for Raymond; thus behavioural experiments were employed as a series of graded exposure tasks [39]. Consequently, the assumption was reframed stepwise and tested gradually, that is, through removal of doorstop, unlocking bedroom door, substituting watching security cameras in the evening with watching movies, and eventually removing barricade on subsequent nights and then every night. Raymond's engagement with social situations, such as casual walks, grocery shopping, and family visits, was encouraged via graded exposure tasks, as well.

Behavioural experiments utilised through graded exposure affected further improvements in Raymond's psychosocial functioning. Tested assumptions were disconfirmed and replaced with* “These are just my habits that are so difficult to break.”* Raymond's safety behaviours relaxed considerably, and some were eventually abandoned.

### 2.7. Cognitive Therapy for Secondary Symptoms

Following completion of cognitive and behavioural interventions for the symptoms of psychosis, Raymond experienced a considerable reduction in his psychological distress and reported low anxiety levels. Therefore, it was no longer necessary for the symptoms of comorbid anxiety to be addressed in a separate module of the intervention.

### 2.8. Self-Management Planning (Relapse Prevention)

The self-management planning focused initially on recognising early warning signs for antecedents of derogatory voices and addressing them accordingly. Organising activities in the evening was already addressed in earlier therapy stages. Hence, prevention of boredom became the focus towards the end of therapy. A family meeting was organised with the community-based mental health team and plans were made for gradual reinstatement of Raymond's interaction with his relatives and acquaintances. Furthermore, during the course of intervention, Raymond reflected on his life and realised that, despite his intellectual capacities and learning potential, he never felt confident enough to undertake further education. Since his abilities to cope with voices and persecutory beliefs increased considerably and anxiety levels reduced, Raymond decided to explore evening courses provided in the local college and pursue further qualifications. Finally, a CBTp self-help guide [40] was introduced to support the client's continuous recovery.

### 2.9. Evaluation of Outcomes

Subjectively, Raymond reported numerous substantial improvements in his psychological functioning at the end of therapy. Some of his pre- and postintervention comments, evidencing subjective importance of the therapy outcomes, are quoted in Table 1.

Not only did the client's strategies to cope with voices, paranoia, and anxiety improved considerably, but also, as illustrated by the pre- and postintervention behaviour frequency samples (Table 2), the second therapeutic goal, that is, reestablishment of autonomy at nights, was achieved as well.

Furthermore, as shown in Table 3, the posttreatment administration of BSI confirmed substantial reduction in both paranoid ideation and anxiety levels, as the client's scores dropped below the UK outpatient means.

## 3. Discussion

The present case report portrays a range of considerable improvements in psychosocial functioning of a person diagnosed with paranoid schizophrenia and comorbid anxiety disorder, following a course of 16 sessions of CBTp, which primarily focused on cognitive restructuring of paranoid appraisals of voices and graded behavioural exposure to anxiety-inducing stimuli. Such outcomes were evaluated by the means of standardised measurements, subjective reflections, and behaviour frequency samples. The intervention outcomes were consistent with previous research findings supporting the effectiveness of CBTp [19–23].

The client did not only arrive at more functional appraisals of his psychosis-type experiences but also achieved a marked improvement in his behavioural functioning, including increased frequency and quality of socially inclusive efforts, reduced withdrawal and isolation, and enhanced functionality of strategies to cope with emotional distress. Such behavioural change seems, again, consistent with the principles and desired goals of both cognitive restructuring and graded exposure in psychosis [32]. In such interventions an individual is helped to reevaluate the validity of his/her problematic anxiety-inducing beliefs that trigger avoidance and inhibit a range of functional reactions, whilst on the other hand the person is assisted with the gradual implementation of desired (approach-type) behaviours that will eventually reinforce more functional and reality-based appraisals of the feared stimuli.

Yet, the original contribution the reported case attempts make to the clinical and research literature concerns the delivery method of CBTp strategies and the particular way through which the substantial psychosocial improvements were achieved, rather than the outcomes themselves. CBTp interventions have traditionally focused on the enhancement of cognitive and behavioural abilities to cope with psychological distress (i.e., secondary/comorbid symptoms, such as anxiety and/or depression) among individuals experiencing psychosis, as a primary therapeutic focus. For instance, as proposed two decades ago by Fowler et al. [41], coping strategies for emotional distress were to be addressed in therapy following the initial assessment and engagement stage. This case report appears to demonstrate that, in some cases, particularly among those individuals that are able to build a functional therapeutic rapport, the actual content of auditory hallucinations and paranoid delusions needs to be directly engaged with, analysed, and collaboratively restructured in order to produce an initial alleviation in comorbid distress. Such alleviation would allow the subsequent behavioural interventions to expose an individual to anxiety-inducing stimuli, encourage interpersonal interaction, and produce a sustained improvement in one's psychosocial functioning.

The reported improvements, however, would not have been achieved without the client's consistent collaboration and engagement, readiness for change, openness to new knowledge, and expressed motivation to overcome his complex and enduring mental health difficulties, which contributed to the development of the functional, helpful, and trusting therapeutic alliance and relationship. The fundamental importance of such client-related factors and their affirmative effects on therapy outcomes have long been acknowledged in research literature [42]. Although the quality and functionality of therapeutic relationship could be considerably affected by the content of paranoid and persecutory beliefs that a person with psychosis may hold about their environment and other people, empirical studies demonstrate that the general levels of clients' satisfaction with CBTp are shown to be high, and the mean client-rated working alliance often appears excellent [21]. Also, clients' acceptability of manualised CBTp has been demonstrated to be stable over time and unaffected by their demographics [42, 43].

In the UK, clinical guidelines, which define the current standards for evidence-based procedures in care and health practice in England and Wales, have at times been criticised for being too prescriptive in their recommendations, thus limiting the clinical judgement of mental health professionals [44]. Yet, as argued by Parry [45], the guidelines serve to provide evidence-based means for an informed clinical judgement rather than substitute such judgement entirely. Also, Mankiewicz and Turner [27] demonstrated that biased perceptions of clinical guidelines might considerably impede the provision levels of empirically embedded interventions in mental health. Thus, it seems imperative that clinicians are aware of successful examples of evidence-based cognitive-behavioural interventions that helped individuals with severe and enduring mental ill-health achieve substantial and often unprecedented improvements in their lives. The case study reported in this paper provides further support for the utilisation of such empirically grounded interventions with individuals experiencing psychosis and comorbid mood disorders, additionally offering an innovative and encouraging perspective on direct engagement with the content of paranoia and auditory hallucinations at the inception of psychological treatment.

## Figures and Tables

**Table 1:** Subjective pre- and postintervention reflections on the patient's own difficulties.

	Preintervention quotes	Postintervention quotes
Voices	*They're scary and they freak me out. *	*They're still there but I don't attend to them as much now. *
Appraisals	*I'm being chased and will be punished. *	*These are probably my own thoughts. *
Anxiety	*I often become scared. Anxiety can be overwhelming. *	*I'm not as anxious as before. I can relax more often now. *
Night time	*The scariest time. *	*I fall asleep easier now. I hardly ever take my sleeping tablets. *
Coping	*I cannot cope. I'm losing control. *	*I can cope and move on. I can see a ray of light in my life now. *

**Table 2:** Self-reported frequency of safety seeking behaviour.

	Preintervention	Postintervention
Setting alarm system	Every night	Irregularly, only some of the nights
Barricading bedroom	Every night	Just closing doors
Watching cameras	Every night	Sometimes briefly during the day/watching movies at night to relax
Hiding at home	Every night/most days	Visiting family, attending outpatient appointments, attending college

**Table 3:** BSI pretreatment assessment and posttreatment evaluation scores for the case.

BSI scale	UK outpatient mean/SD	Pretreatment score	Posttreatment score
PAR	M = 1.54/SD = 1.08∗	2.20	1.50
ANX	M = 1.87/SD = 1.03∗	2.50	1.60

*Reported by Ryan [31].
